# Corrigendum to ‘A novel multiplex qPCR assay for detection of Plasmodium falciparum with histidine-rich protein 2 and 3 (pfhrp2 and pfhrp3) deletions in polyclonal infections’

**DOI:** 10.1016/j.ebiom.2021.103261

**Published:** 2021-02-24

**Authors:** Lynn Grignard, Debbie Nolder, Nuno Sepulveda, Araia Berhane, Selam Mihreteab, Robert Kaaya, Jody Phelan, Kara Moser, Donelly A. van Schalkwyk, Susana Campino, Jonathan B. Parr, Jonathan J. Juliano, Peter Chiodini, Jane Cunningham, Colin J. Sutherland, Chris Drakeley, Khalid B. Beshir

**Affiliations:** a Faculty of Infectious Diseases, London School of Hygiene and Tropical Medicine, United Kingdom; b PHE Malaria Reference Laboratory, London School of Hygiene & Tropical Medicine, United Kingdom; c Centre of Statistics and Applications of University of Lisbon, Portugal; d Communicable Diseases Control Division, Ministry of Health, Eritrea; e Kilimanjaro Christian Medical University College, Tanzania; f University of North Carolina at Chapel Hill, United States; g UCL Hospital for Tropical Diseases, United Kingdom; h World Health Organization, Geneva, Switzerland

The authors wish to correct two typographical errors in the manuscript. In the Methods (Section 5.3: Assay optimization), the concentration unit of dNTPs was wrongly written as 800 nM (nanomolar) and should be corrected to 800mM (millimolar). Furthermore, in Table S1 of the Supplementary material, the primers and probe sequences for *Pfhrp3* are incorrect. They should be written: *Pfhrp3*_F2: 5’-ACGGATTTCATTTTAACCCTTCACGA-‘3, *Pfhrp3*_R2: 5’-TGAGAATCATCAAAACAAGCATTAGC-‘3 and *Pfhrp3*_probe: JOE’-ACAATTCCCATACTTTACATCATGCA-‘3 BHQ1. A revised Table S1 is included (below). The primers and probe sequences of *Pfhrp3* in Figure 3S of the supplementary material are correct. The authors regret any confusion caused and appreciate the opportunity to correct these mistakes

**Table S1:** **Primers used for the three parasite target genes and one human gene.** Pfhrp2_R2 primer was modified at 3' end (T->G, highlighted) to increase specificity. Final primer used in the optimized experiments are highlighted in bold.

**Name**	**Primer sequence**	**Reference**
**Pfhrp2_F1**	5’ TAATTSCGYATTTAATAATAACTTGTG-‘3	This study
Pfhrp2_F2	5’-TAATTCCGCATTTAATAATAAC**G**TGTG-3’	
Pfhrp2_F3	5’-TAATTCCGCATTTAATAATAAC**G**TT**G**G-3’	
Pfhrp2_R1	5’- CATCATCTACATGTGCTTGAG -‘3	
**Pfhrp2_R2**	5’- CATCATCTACATGTGCT**G**GAG -‘3	
Pfhrp2_R3	5’- CATCATCTACATGTGC**G**TGAG -‘3	
**Pfhrp2_probe**	FAM 5’-ATGCAAAAGGACTTAATTTAAATAAGAGATT-‘3 BHQ2	
Pfhrp3_F1	5’-TCCGAATTTAACAATAACTTGTTTAGC-‘3	
Pfhrp3-R1	5’-GTCAAGCACATGCAGGTGATG-‘3	
Pfhrp3_P1	JOE 5’-ATGCAAAAGGACTTAATTCAAATAAGAGATTA-‘3 BHQ1	
**Pfhrp3_F2**	5’-ACGGATTTCATTTTAACCCTTCACGA-‘3	
**Pfhrp3_R2**	5’-TGAGAATCATCAAAACAAGCATTAGC-‘3	
**Pfhrp3_probe**	JOE’-ACAATTCCCATACTTTACATCATGCA-‘3 BHQ1	
**Pfldh_F**	5’-ACGATTTGGCTGGAGCAGAT-‘3	[1]
**Pfldh_R**	5’-TCTCTATTCCATTCTTTGTCACTCTTTC-‘3	
**Pfldh_probe**	ROX 5’-GTAATAGTAACAGCTGGATTTACCAAGGCCCCA-‘3 BHQ1	
**HumTuBB_F**	5’-AAGGAGGTCGATGAGCAGAT-‘3	[2]
**HumTuBB_R**	5’-GCTGTCTTGACATTGTTGGG-‘3	
**HumanTuBB_P**	CY5 5’-TTAACGTGCAGAACAAGAACAGCAGCT-‘3 BHQ2	

The authors would like to apologise for any inconvenience caused.
