# Supplementary material for: A novel multiplex qPCR assay for detection of Plasmodium falciparum with histidine-rich protein 2 and 3 (pfhrp2 and pfhrp3) deletions in polyclonal infections
Source: eBioMedicine. 2020 May 8;55:102757. doi: 10.1016/j.ebiom.2020.102757 (PMC7218259; doi:10.1016/j.ebiom.2020.102757)
Supplement: Supplementary file 1 [file mmc1.docx]

**Supplementary Material**

**A Novel Multiplex qPCR Assay for Detection of *Plasmodium* *falciparum* with *Histidine-rich Protein 2 and 3 (pfhrp2 and pfhrp3)* Deletions in Polyclonal Infections**

Lynn Grignard^1^, Debbie Nolder^1,2^, Nuno Sepúlveda^1,3^, Araia Berhane^4^, Selam Mihreteab^4^, Robert Kaaya^5^, Jody Phelan^1^, Kara Moser^6^, Donelly A. van Schalkwyk^1^, Susana Campino^1^, Jonathan Parr^6^, Jonathan J. Juliano^6^, Peter Chiodini^1,7^, Jane Cunningham^8^, Colin J. Sutherland^1^, Chris Drakeley^1^ and Khalid B. Beshir^1^*

1. Faculty of Infectious Diseases, London School of Hygiene and Tropical Medicine, United Kingdom
2. PHE Malaria Reference laboratory, London School of Hygiene & Tropical Medicine, United Kingdom
3. Centre of Statistics and Applications of University of Lisbon, Portugal
4. Communicable Diseases Control Division, Ministry of Health, Eritrea
5. Kilimanjaro Christian Medical University College, Tanzania
6. University of North Carolina, United States
7. UCL Hospital for Tropical Diseases, United Kingdom
8. World Health Organization, Geneva, Switzerland

**Key words:** *pfhrp2, pfldh,* qPCR, RDT, malaria

*Corresponding author: [Khalid.Beshir@lshtm.ac.uk](mailto:Khalid.Beshir@lshtm.ac.uk)

**Supplementary Material: Figures and Tables**


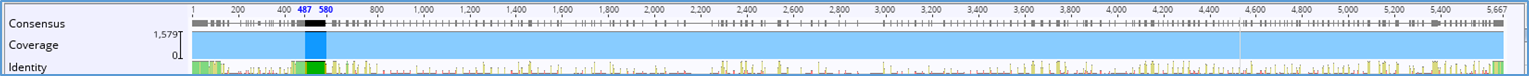


**Figure S1: Location of *pfhrp2* primers and probe: consensus sequence of *pfhrp2* achieved by multiple sequence alignment of 1581 samples obtained from MalariaGEN.**


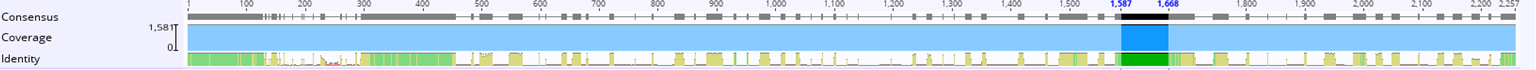


**Figure S2: Location of *pfhrp3* primers and probe: consensus sequence of *pfhrp3* achieved by multiple sequence alignment of 1581 samples obtained from MalariaGEN.**

**Figure 3S: Multiple alignment of *pfhrp2* and *pfhrp3*:** The two genes were aligned to check primer cross binding. Two nucleotide (bold) of the 3’ end of the *pfhrp2* reverse primer were modified to increase specificity. *Pfhrp2* reverse primer already has one nucleotide difference with *pfhrp3* at the 3’ end and this was exploited in the design.

**Table S1: Primers used for the three parasite target genes and one human gene.** *Pfhrp2_R2* primer was modified at 3’ end (T->G, highlighted) to increase specificity. Final primer used in the optimized experiments are highlighted in bold.

| Name | Primer sequence | Reference |
| --- | --- | --- |
| Pfhrp2_F1 | 5’ TAATTSCGYATTTAATAATAACTTGTG-‘3 | This study |
| Pfhrp2_F2 | 5’-TAATTCCGCATTTAATAATAAC**G**TGTG-3’ |  |
| Pfhrp2_F3 | 5’-TAATTCCGCATTTAATAATAAC**G**TT**G**G-3’ |  |
| Pfhrp2_R1 | 5’- CATCATCTACATGTGCTTGAG -‘3 |  |
| Pfhrp2_R2 | 5’- CATCATCTACATGTGCT**G**GAG -‘3 |  |
| Pfhrp2_R3 | 5’- CATCATCTACATGTGC**G**TGAG -‘3 |  |
| Pfhrp2_probe | FAM 5’-ATGCAAAAGGACTTAATTTAAATAAGAGATT-‘3 BHQ2 |  |
| Pfhrp3_F1 | TCCGAATTTAACAATAACTTGTTTAGC |  |
| Pfhrp3-R1 | GTCAAGCACATGCAGGTGATG |  |
| Pfhrp3_P1 | ATGCAAAAGGACTTAATTCAAATAAGAGATTA |  |
| Pfhrp3_F2 | 5’-TCCGAATTTAACAATAACTTGTTTAGC-‘3 |  |
| Pfhrp3_R2 | 5’-GTCAAGCACATGCAGGTGATG-‘3 |  |
| Pfhrp3_probe | JOE 5’-ATGCAAAAGGACTTAATTCAAATAAGAGATTA-‘3 BHQ1 |  |
| Pfldh_F | 5’-ACGATTTGGCTGGAGCAGAT-‘3 | (1) |
| Pfldh_R | 5’-TCTCTATTCCATTCTTTGTCACTCTTTC-‘3 |  |
| Pfldh_probe | ROX 5’-GTAATAGTAACAGCTGGATTTACCAAGGCCCCA-‘3 BHQ1 |  |
| HumTuBB_F | 5’-AAGGAGGTCGATGAGCAGAT-‘3 | (2) |
| HumTuBB_R | 5’-GCTGTCTTGACATTGTTGGG-‘3 |  |
| HumanTuBB_P | CY5 5’-TTAACGTGCAGAACAAGAACAGCAGCT-‘3 BHQ2 |  |

**Figure S4. Initial experiment using initial *pfhrp2* (left) and *pfhrp3* (right) primers (first panel); after changing *pfhrp3* target sequence (middle panel) and after modification of *pfhrp2* reverse primer (bottom panel)**

**
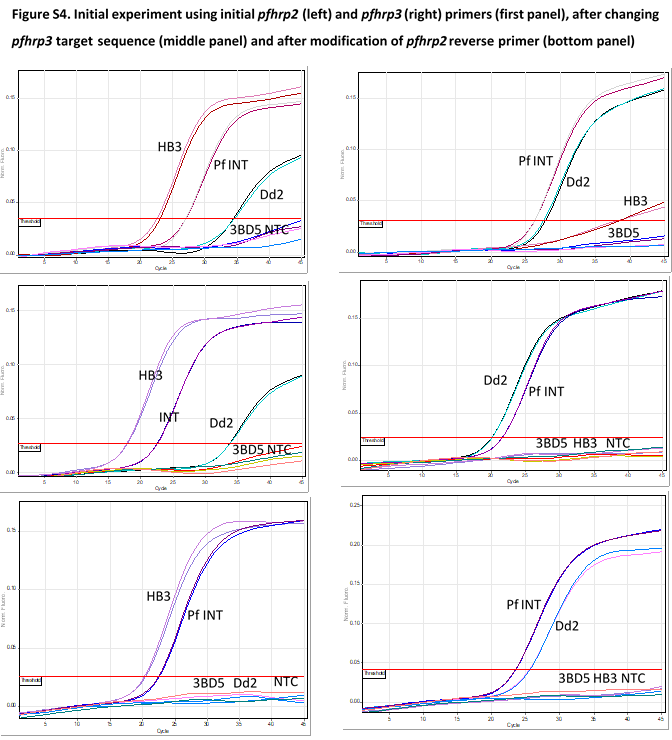
**

**Table S2. Quantification cycle (C_q_) produced by different primer combinations.** The best C_q_ value and negative results in 3BD5 and Dd2 was obtained when *pfhrp2*_F1 and *pfhrp2*_R2 were combined. SD, standard deviation.

|  | Mean C_q_ value (SD) | | | | | | | | | | | | |
| --- | --- | --- | --- | --- | --- | --- | --- | --- | --- | --- | --- | --- | --- |
|  | | ***Pfhrp2_F1*** | | | | *Pfhrp2_F2* | | | | *Pfhrp2_F3* | | | |
|  | | **C_q_** | | **SD** | | **C_q_** | **SD** | | | **C_q_** | **SD** | | |
| *Pfhrp2_R1* | 3BD5 | NEG |  | |  | | |  | NEG | | |  |  |
|  | Dd2 | 42.8 | 0.31 | | 32.81 | | | 0.19 | NEG | | |  |  |
|  | HB3 | 29.5 | 0.11 | | 22.54 | | | 0.13 | 29.15 | | | 0.71 |  |
|  | pfINT | 30.64 | 0.13 | | 23.40 | | |  | 28.04 | | | 0.17 |  |
|  | NTC | NEG |  | | NEG | | |  | NEG | | |  |  |
| *Pfhrp2_R2* | 3BD5 | **NEG** |  | | NEG | | |  | NEG | | |  |  |
|  | Dd2 | **NEG** |  | | 34.24 | | | 0.23 | NEG | | |  |  |
|  | HB3 | **21.08** | 0.06 | | 23.53 | | | 0.50 | 31.35 | | | 0.64 |  |
|  | pfINT | **22.14** | 0.10 | | 24.65 | | | 0.21 | 30.03 | | | 0.023 |  |
|  | NTC | **NEG** |  | | NEG | | |  | NEG | | |  |  |
| *Pfhrp2_R3* | 3BD5 | NEG |  | | NEG | | |  | NEG | | |  |  |
|  | Dd2 | 33.85 | 0.34 | | 31.24 | | | 0.12 | NEG | | |  |  |
|  | HB3 | 22.17 | 0.12 | | 20.98 | | | 0.38 | 27.25 | | | 0.04 |  |
|  | pfINT | 23.16 | 0.17 | | 22.32 | | | 0.22 | 27.80 | | | 0.12 |  |
|  | NTC | NEG |  | | NEG | | |  | NEG | | |  |  |

**Table S3. Robustness and precision of the qPCR assay:** Each assay in the qPCR detected the 8 replicates each of the Pf INT 3 and 1.5 parasites per µl and showed no amplification in the 20 replicates of pf-negative DNA blood.

| Mean C_q_ values | | | | | | | | | | | | |
| --- | --- | --- | --- | --- | --- | --- | --- | --- | --- | --- | --- | --- |
| Parasite  Per µl | ***pfhrp2*** | | ***pfhrp3*** | | | | ***pfldh*** | | | | | |
|  | **Mean** | **SD** | **Mean** | | **SD** | | **CV** | | **Mean** | | **SD** | |
| 3 | 35.56 | 0.57 | 34.81 | | 0.59 | | 1.91 | | 35.12 | | 0.58 | |
| 1.5 | 36.77 | 0.80 | 37.28 | | 0.71 | | 1.90 | | 36.69 | | 0.74 | |
| 0.76 | 38.76 | 1.49 | 38.55 | | 1.37 | | 3.56 | | 38.35 | | 1.48 | |
| 0.38 | 40.73 | 1.68 | 40.36 | | 1.67 | | 4.13 | | 40.01 | | 1.60 | |
|  |  |  |  | |  | |  | |  | |  | |
| Estimated parasite density (parasite per µl) | | | | | | | | | | | | |
| Parasite  Per µl | ***pfhrp2*** | | | ***pfhrp3*** | | | | | | ***pfldh*** | | |
|  | **Mean** | **SD** | **CV** | **Mean** | | **SD** | | **CV** | | **Mean** | **SD** | **CV** |
| 3 | 2.86 | 0.24 | 8.34 | 2.95 | | 0.29 | | 9.81 | | 2.62 | 0.23 | 8.83 |
| 1.5 | 0.98 | 0.63 | 32.63 | 0.57 | | 0.16 | | 26.12 | | 0.95 | 0.25 | 28.12 |
| 0.76 | 0.21 | 0.46 | 109.15 | 0.20 | | 0.22 | | 98.80 | | 0.26 | 0.26 | 107.91 |
| 0.38 | 0.05 | 0.12 | 114.77 | 0.06 | | 0.07 | | 114.14 | | 0.07 | 0.08 | 112.26 |

**Table S4. Limit of detection and Limit of quantification of the qPCR assay:** the lowest parasite density (3 parasite per µl) positive in the standard dilution was further diluted 2-fold until 0.38 parasite per µl. The lowest diluted sample that can be detected with CV of parasite density of 35% lies between 1.5 and 0.76 parasites per µl.

| **Sample** | ***pfhrp2*** | | ***pfhrp3*** | | ***pfldh*** | | | ***humTuBB*** | | |
| --- | --- | --- | --- | --- | --- | --- | --- | --- | --- | --- |
|  | **Mean** | **SD** | **Mean** | **SD** | **Mean** | **SD** | **Mean** | | **SD** |  |
| **INT 3 p/ul** | 34.12 | 0.54 | 35.09 | 0.76 | 34.39 | 0.36 | 20.09 | | 0.18 |  |
| **Pf INT 1.5 p/ul** | 36.57 | 0.82 | 36.96 | 0.73 | 36.83 | 0.75 | 19.73 | | 0.15 |  |
| **pf-negative DNA blood** | NEG |  | NEG |  | NEG |  | 20.1 | | 0.24 |  |

**Table S5. Percentage of *pfhrp2*- and *pfhrp3*-deleted clones in artificially mixed laboratory isolate 3BD5 and Pf INT.** The highest and lowest parasite densities in the mixture was 1.5x10^5^ and 1.5 parasite per µl respectively and this represents the 1:100000 and 100000:1 ratios.

| **3BD5: Pf INT ratio** | *pfhrp2 deletion* % | | | *pfhrp3 deletion* % | | |
| --- | --- | --- | --- | --- | --- | --- |
|  | Mean | 95% CI | | Mean | 95% CI | |
| **1:1** | 53.18 | 52.41 | 53.96 | 59.23 | 57.65 | 60.80 |
| **5:1** | 81.90 | 80.84 | 82.97 | 81.31 | 77.08 | 85.55 |
| **10:1** | 91.78 | 91.38 | 92.18 | 92.36 | 90.43 | 94.29 |
| **100:1** | 98.67 | 98.59 | 98.75 | 99.20 | 99.17 | 99.23 |
| **1000:1** | 99.91 | 99.91 | 99.92 | 99.91 | 99.84 | 99.99 |
| **10000:1** | 99.98 | 99.98 | 99.99 | 99.97 | 99.97 | 99.98 |
| **100000:1** | 99.98 | 99.96 | 100.01 | 99.99 | 99.99 | 99.99 |
| **1:1** | 53.92 | 50.86 | 56.97 | 49.65 | 48.52 | 50.77 |
| **1:5** | 17.62 | 9.82 | 25.42 | 18.65 | 16.73 | 20.57 |
| **1:10** | 9.97 | 7.41 | 12.53 | 16.14 | 14.06 | 18.2 |
| **1:100** | 1.06 | 1.01 | 1.12 | 1.29 | 0.78 | 1.80 |
| **1:1000** | 1.07 | 1.00 | 1.15 | 8.05 | 7.81 | 8.30 |
| **1:10000** | 1.86 | 1.56 | 2.16 | 0.14 | 0.06 | 0.22 |
| **1:100000** | 3.27 | 3.16 | 3.38 | 1.29 | 1.14 | 1.43 |

**Table S6: *In silico* analysis of recently published *pfhrp2* and *pfhrp3* qPCR primers and probes**: The sequence analysis was carried out by multiple alignment of *pfhrp2* and *pfhrp3* sequences from 1581 published *P.* *falciparum* genomes (MalariaGEN) from Africa, SE Asia and south America. The recently published qPCR primers and probes (3, 4) were searched in the database and mutations (highlighted), insertions (underlined) and deletions (strikethrough) within primer/probe are reported. ^*^ Country where mutations were found. ^**^ Both reverse primers showed partial binding to the variable region of *pfhrp2* and *pfhrp3*.

| **Reference** | **Oligo name** | **Sequence** | **Country ^*^** |
| --- | --- | --- | --- |
| (4) | *hrp2*  reverse^**^ | GCTACATGATGAGCATGA  GCTACATG~~ATGAGCATG~~A  GCTACATGGTGAGCATGATGAGCATGA  GCTGCATGATGTACATGATGAGCATGA GCTACATGATGGGCATCGGCAACATGATGAGCATGA GCTGCATGATGGGCATCGGCTACATGATGAGCATGA | DRC, Ghana, Laos, Malawi, Thailand and Vietnam |
|  | *hrp3*  forward^**^ | AGGACTTAATTCAAATAAGAGATTA | Ghana, Guinea, Malawi and Thailand |
| *(3)* | *pfhrp2* forward | GTATTATCCGCTGCCGTTTTTGCC | Ghana |
|  | *pfhrp2*  reverse | TCTACATGTGCTTGAGTTTCG | Bangladesh, Cambodia, Laos and Vietnam |
|  | *pfhrp2*  probe | TTCCGCATTTAATAATAACTTGTGTAGC | The Gambia, Ghana and Mali |
|  | *pfhrp3* forward | ATATTATCCGCTGCCGTTTTTGCT | Malawi |
|  | *pfhrp3*  probe | CTCCGAATTTAACAATAACTTGTTTAGC | Bangladesh, Ghana, Guinea, Malawi and Mali |

**Supplementary material**

**References**

1. Parr JB, Verity R, Doctor SM, Janko M, Carey-Ewend K, Turman BJ, et al. Pfhrp2-Deleted Plasmodium falciparum Parasites in the Democratic Republic of the Congo: A National Cross-sectional Survey. J Infect Dis. 2017;216(1):36-44.

2. Beshir KB, Hallett RL, Eziefula AC, Bailey R, Watson J, Wright SG, et al. Measuring the efficacy of anti-malarial drugs in vivo: quantitative PCR measurement of parasite clearance. Malar J. 2010;9:312.

3. Schindler T, Deal AC, Fink M, Guirou E, Moser KA, Mwakasungula SM, et al. A multiplex qPCR approach for detection of pfhrp2 and pfhrp3 gene deletions in multiple strain infections of Plasmodium falciparum. Sci Rep. 2019;9(1):13107.

4. Kreidenweiss A, Trauner F, Rodi M, Koehne E, Held J, Wyndorps L, et al. Monitoring the threatened utility of malaria rapid diagnostic tests by novel high-throughput detection of Plasmodium falciparum hrp2 and hrp3 deletions: A cross-sectional, diagnostic accuracy study. EBioMedicine. 2019;50:14-22.
